# Ultrasound-guided haemodynamic profiling in haemodialysis patients: a single-centre prospective observational study protocol

**DOI:** 10.3389/fneph.2026.1939088

**Published:** 2026-10-20

**Authors:** Samuel Larsen, Fabio Silvio Taccone, Alain Le Moine, Filippo Annoni, Thomas Baudoux

**Affiliations:** 1 Department of Nephrology, HUB Hôpital Erasme, Université libre de Bruxelles, Brussels, Belgium; 2 Department of Intensive Care, Hôpital Universitaire de Bruxelles (HUB), Université libre de Bruxelles, Brussels, Belgium

**Keywords:** cardionephrology, fluid management, haemodialysis, intradialytic hypotension, lung ultrasound, point-of-care ultrasound, VExUS

## Abstract

**Background:**

Intradialytic hypotension (IDH) and chronic fluid overload are common and clinically important complications of maintenance haemodialysis, yet routine volume assessment remains imprecise. Multi-organ point-of-care ultrasound may provide a structured bedside assessment of cardiac reserve, pulmonary congestion and venous congestion.

**Methods and analysis:**

This single-centre prospective observational cohort study will enrol approximately 60 adult patients receiving maintenance haemodialysis. Participants will undergo integrated ultrasound profiling immediately before and after dialysis at baseline and at 3, 6 and 9 months, with clinical outcomes followed for 12 months. The ultrasound protocol includes focused cardiac ultrasound, eight-zone lung ultrasound B-line quantification and a modified VEXUS assessment using inferior vena cava, hepatic vein and portal vein findings. Event-triggered intradialytic scans may also be performed for current IDH or suspected mismatch between the prescribed target weight and the best clinically tolerated weight. These examinations will not be pooled with scheduled assessments. A separate exploratory post-scan association analysis will include only scans completed while the patient is clinically stable, before the first same-session IDH and not in response to evolving current-session haemodynamic instability; the outcome is subsequent IDH before session end. Scans obtained during or after IDH or during evolving instability will be described only. The main descriptive objective is to characterise pre- and post-dialysis ultrasound-derived haemodynamic profiles and their within-session and longitudinal variability. The primary prespecified analysis for Objective 2 is an exploratory and non-confirmatory assessment of the within-patient association between pre-dialysis ultrasound parameters and same-session IDH using a scheduled-session case-crossover design. Secondary analyses include the separate event-triggered post-scan association analysis and an exploratory scheduled-session mixed-effects analysis. Event-triggered examinations will not contribute cases to the primary analysis. Exploratory objectives assess clinician-perceived usefulness and documented clinician-implemented prescription changes after delayed disclosure, without assessing clinical benefit.

**Ethics and dissemination:**

The study has received ethics approval and will be conducted after written informed consent. Results will be disseminated through peer-reviewed publications and scientific meetings.

**Study registration:**

https://www.isrctn.com/ISRCTN80571205, identifier ISRCTN80571205.

## Introduction

Patients with end-stage renal disease on haemodialysis (HD) face a delicate balance in fluid management. Haemodynamic instability during HD, particularly intradialytic hypotension (IDH), is a frequent and serious complication of chronic HD (1). Reported IDH prevalence ranges from 8% to 40% across studies and definitions. IDH is associated with distress, repeated end-organ ischaemia and vascular access thrombosis (1); large observational data also associate IDH with cardiovascular events and all-cause mortality (2). Conversely, chronic fluid overload between dialysis sessions contributes to hypertension, heart failure and pulmonary oedema (3) and is independently associated with mortality across pre-dialysis blood-pressure categories (4). Achieving and maintaining an optimal “dry weight” is therefore crucial, as both excessive fluid removal and volume expansion can precipitate adverse events (3). However, accurately assessing volume status and perfusion in HD patients remains challenging. Traditional methods - including clinical examination, blood pressure trends, weight changes and chest X-rays - have important limitations for assessing volume status and ultrafiltration tolerance (3). As a result, clinicians often lack real-time, objective measures to guide ultrafiltration to prevent IDH or fluid overload.

Rationale for an Integrated Ultrasound Approach: Advances in point-of-care ultrasonography offer a potential solution by directly visualising cardiovascular and volume status. Cardiac ultrasound (echocardiography) can evaluate cardiac function and filling pressures; in patients with preserved ejection fraction, dynamic septal E/e’ patterns differed between those with and without IDH, and lower septal E/e’ was independently associated with IDH (5). Lung ultrasound allows bedside detection of extravascular lung water via B-lines - vertical artefacts that increase in pulmonary oedema. In critically ill adults, lung-ultrasound B-line scores have correlated with invasively measured extravascular lung water index, supporting their use as a non-invasive marker of pulmonary congestion (6). Venous ultrasound, using the Venous Excess Ultrasound (VEXUS) framework, assesses systemic venous congestion by combining inferior vena cava (IVC) size with Doppler waveforms of the hepatic, portal and intrarenal veins (7). The original composite VEXUS score grades the severity of venous congestion; for example, a dilated IVC (≥2 cm) plus severely abnormal hepatic and portal venous flow patterns can indicate severe congestion (7). This technique assesses venous congestion from the organ’s perspective rather than serving as a direct gauge of intravascular volume, and forms part of the broader concept of fluid tolerance (8). Dialysis-specific data also suggest that these domains provide complementary information: in an 18-participant pilot study, B-line scores were not consistently correlated with IVC indices, bioimpedance or echocardiographic measures, suggesting that they reflect partly distinct aspects of haemodynamic status (9). Together, these measurements provide a bedside assessment of cardiac reserve, pulmonary congestion and systemic venous congestion. Such a multi-organ point-of-care ultrasound approach may better identify patients at risk of IDH, for example those with limited cardiac reserve or high venous pressures, and may ultimately support safer ultrafiltration decisions than one-dimensional measures such as blood pressure or weight change.

Existing Evidence and Gaps: Early studies support the feasibility and potential utility of ultrasound-based haemodynamic assessment in dialysis patients, though significant gaps remain. Chen et al. reported associations between dynamic E/e’ changes and IDH during haemodialysis (5). In a prospective single-session study of 107 patients, Allinovi et al. found that lower pre-dialysis B-line count was independently associated with same-session IDH; unlike the present protocol, however, the analysis compared patients rather than repeated sessions within patients (10). In a separate multicentre cohort of 392 patients, ultrasound-detected pulmonary congestion was frequently asymptomatic or only mildly symptomatic and independently predicted mortality and cardiac events (11). Several small prospective studies have applied VEXUS and lung ultrasound in the haemodialysis setting. Leyba et al. reported in a two-centre cohort that large-volume fluid removal by haemodialysis produced measurable improvements in VEXUS grade and lung-ultrasound B-line count from pre- to post-dialysis, indicating that these measures capture dynamic volume-related change (12). In the ACUVEX study, serial VEXUS and lung-ultrasound examinations were performed during haemodialysis: approximately 15% of patients had an elevated VEXUS grade at baseline, and VEXUS grade decreased after ultrafiltration in those patients (13). All participants with an elevated VEXUS grade in that study had biventricular systolic dysfunction, suggesting that VEXUS-positive congestion in haemodialysis may be closely linked to underlying cardiac dysfunction rather than volume overload alone (13). Tonelli et al. observed that portal-vein Doppler pulsatility was particularly responsive to volume removal, whereas changes in IVC diameter and hepatic-vein Doppler were more modest (14). Conversely, Aslaner et al. found that VEXUS did not discriminate the need for emergency haemodialysis among maintenance haemodialysis patients evaluated in the emergency department; portal-vein Doppler showed only modest discrimination (15). These findings support further evaluation but do not justify using ultrasound congestion markers as standalone clinical decision tools. Standardised protocols for integrating these tools into dialysis care remain to be established, and prospective evaluation of patient-centred outcomes is still needed (3). A recent review concluded that lung ultrasound, focused echocardiography and VEXUS are promising tools for dialysis fluid management, while emphasising the need for standardisation and prospective evaluation of clinical outcomes (3). Randomised haemodialysis-specific evidence supports the clinical relevance of B-line assessment, although effects on patient-centred outcomes remain uncertain. In an earlier 71-patient trial, lung-ultrasound-guided dry-weight reduction reduced B-line burden and ambulatory blood pressure over eight weeks; the proportion of patients experiencing at least one IDH episode was not higher in the intervention group (16). In the LUST trial, Zoccali et al. achieved more frequent relief of pulmonary congestion than usual care, but did not improve the prespecified composite clinical outcome (17). More recently, Kaysi et al. found that a simplified post-dialysis eight-zone B-line-guided dry-weight strategy reduced B-line burden over 60 days without a statistically significant increase in IDH; however, that trial did not assess major clinical outcomes (18).

## Methods and analysis

### Study objectives

Study Objective: We propose a single-centre prospective observational cohort study to evaluate an ultrasound-based haemodynamic profiling strategy in chronic HD patients. The parent cohort will provide serial, integrated assessments of cardiac function, lung water, and venous congestion at quarterly intervals and generate a dataset from which several pre-planned analytic objectives will be addressed.

This protocol defines four prespecified objectives within one parent cohort. Each objective has distinct outcomes and analyses. Objectives 1 and 2 are the main study objectives: Objective 1 is descriptive, whereas Objective 2 is the primary prespecified but exploratory and non-confirmatory association analysis for IDH. Objectives 3 and 4 are explicitly exploratory.

- Objective 1 - Descriptive characterisation of ultrasound-derived haemodynamic profiles. Aim: to characterise the magnitude and variability of pre- and post-dialysis ultrasound parameters (cardiac, lung, VEXUS) across sessions and over time, including within-session changes with ultrafiltration and between-session trajectories. Status: main descriptive objective.
- Objective 2 - Ultrasound profile and intradialytic hypotension: within-patient analyses. Aim: to estimate the within-patient association between pre-dialysis ultrasound parameters and same-session IDH, using an embedded case-crossover design restricted to scheduled profiled sessions, with each patient serving as their own control. In practical terms, this analysis compares sessions with IDH to sessions without IDH within the same patient, asking whether that patient’s pre-dialysis ultrasound profile differed before unstable versus stable sessions. Event-triggered examinations will not be pooled with scheduled examinations or contribute to the primary case-crossover. A separate exploratory post-scan association analysis will assess whether ultrasound parameters measured during clinically stable intradialytic scans completed before the first same-session IDH are associated with subsequent IDH before session end. Scans obtained during or after IDH or during evolving haemodynamic instability will be used only to describe the peri-IDH haemodynamic phenotype. A further secondary analysis will estimate the session-level association between pre-dialysis ultrasound parameters and IDH using a generalised linear mixed model with a patient-level random intercept. Status: primary prespecified analysis for Objective 2; exploratory and non-confirmatory.
- Objective 3 - Clinician-directed prescription changes after ultrasound disclosure. Aim: to estimate the proportion of scheduled profiled sessions in which post-session disclosure of ultrasound findings results in a prescription change entered in the dialysis prescription program, and to describe perceived usefulness and confidence. Status: exploratory (descriptive).
- Objective 4 - Longitudinal clinical outcomes. Aim: to explore the association between serial ultrasound trajectories and time-to-event outcomes (hospitalisation, cardiovascular events, mortality). Status: exploratory and hypothesis-generating, with expected event counts insufficient for confirmatory analysis.

Each analytic objective may generate separate publications. The pre-specification of objectives, analyses, and evidentiary status is intended to prevent *post-hoc* selection from a broad exploratory dataset and to allow reviewers to evaluate each objective on its own terms.

### Study design and setting

This is a single-centre, prospective observational cohort study of adult patients undergoing maintenance haemodialysis. We plan to enrol approximately 60 patients from our outpatient HD unit. Each participant will undergo serial ultrasound assessments before and after a dialysis session at 3-month intervals over the study period (at baseline, 3, 6, and 9 months), as detailed in **Table 1**. Recruitment is planned to start in July 2026, and each participant will be followed for 12 months.

**Table 1:** Participant timeline and schedule of study procedures.

Procedure	Screening/Enrolment	Baseline (Visit 1)	Month 3 (Visit 2)	Month 6 (Visit 3)	Month 9 (Visit 4)	Event-triggered (*ad hoc*)
Informed consent	X					
Eligibility screening	X					
Baseline demographics/comorbidities		X				
Pre-dialysis POCUS (cardiac, lung, VEXUS)		X	X	X	X	
Post-dialysis POCUS (cardiac, lung, VEXUS)		X	X	X	X	(if feasible)
Event-triggered intradialytic POCUS						X^a^
Ultrasound disclosure to treating physician		X^b^	X^b^	X^b^	X^b^	X^b^
Clinician pre and post-disclosure questionnaire and documentation of implemented prescription changes		X	X	X	X	(if feasible)
Session-level clinical data (CRF)^c^		X	X	X	X	X
IDH event documentation by nursing staff		X	X	X	X	X
Intradialytic BP monitoring: pre-dialysis, every 30 minutes, end of dialysis and additionally when clinically indicated		X	X	X	X	X
Laboratory values (routine clinical)		X	X	X	X	
Clinical outcome tracking (hospitalisations, CV events, mortality)		Continuous follow-up	Continuous follow-up	Continuous follow-up	Continuous follow-up	

^a^ Event-triggered POCUS may be performed when clinically feasible and safe for current IDH, suspected clinically significant fluid overload or suspected underestimated dry weight, corresponding to a prescribed target weight suspected to be too high or too low, respectively. All event-triggered examinations are intradialytic/intermediate. For the separate post-scan association analysis, scan completion defines the index time and eligibility requires a clinically stable scan completed before the first same-session IDH and not in response to evolving current-session haemodynamic instability. Scans obtained during or after IDH or during evolving instability will be used only to describe the peri-IDH haemodynamic phenotype. Trigger indication is described but does not define separate inferential subgroups.

^b^ Disclosure occurs only after the dialysis session is complete and all intradialytic outcome data have been recorded; exception: findings meeting the emergency disclosure criteria are communicated immediately. Any physician-selected change after routine disclosure is entered in the dialysis prescription program and applies only after the completed index session.

^c^ Session-level CRF captures routinely available session variables where recorded, including pre-dialysis BP and heart rate, weight change, ultrafiltration data, prescribed dialysis-session characteristics, IDH events/interventions and, for event-triggered scans, indication, time from dialysis initiation to scan completion, scan-time BP and ultrafiltration, recent IDH history, temporal relation to the first same-session IDH and derived planned dialysis time remaining.

To capture both peak volume load and more steady-state conditions, participants will be allocated at enrolment to have their scheduled study ultrasound performed either during the first dialysis session after the long interdialytic interval (2/5 of participants) or during a mid-/late-week session (3/5 of participants). This allocation will be prespecified and kept consistent for each participant across all scheduled follow-up timepoints. Session type (post-long interdialytic interval vs mid-/late-week) will be recorded for every visit.

Embedded case-crossover substudy (Objective 2). An embedded within-patient case-crossover design will compare pre-dialysis ultrasound parameters at sessions where IDH occurs with the same patient’s sessions where IDH does not occur. The primary case-crossover analysis is restricted to scheduled profiled sessions, thereby ensuring that all ultrasound measurements are obtained before the dialysis session begins and that the estimand retains a predictive interpretation. Because control sessions are drawn from the same patient’s own scheduled profiled sessions that were free of IDH, all time-invariant between-patient confounders (age, sex, comorbidities, dialysis vintage and vascular access type) are controlled by design. Each participant’s scheduled study sessions remain on the same assigned session type throughout follow-up; case and control sessions in the primary scheduled analysis are therefore inherently aligned on session type. Session timing will be recorded and reported, and the interval between case and control sessions will be considered in sensitivity analyses. The scheduled-session analysis remains primary because it directly addresses the central within-patient question and preserves temporal ordering; its primary status reflects the prespecified analytic hierarchy rather than confirmatory statistical power.

Key time-varying covariates that differ between sessions and are not inherently controlled by the within-patient design - including interdialytic weight gain, pre-dialysis blood pressure, prescribed ultrafiltration volume, dialysate sodium, calcium and temperature, and prescribed session duration - will be considered in adjusted analyses when they are available and reliably recorded in the case report form. Serial measurements from participants who remain free of IDH throughout the study will be used to describe temporal stability and within-patient variability in ultrasound measurements; this will not constitute an empirical test of the case-crossover exchangeability assumption. Adjusted case-crossover analyses will be undertaken only if supported by the number of outcome-informative patient strata and the within-patient variability of the relevant ultrasound parameters; otherwise, analyses will be univariable or minimally adjusted.

Event-triggered substudy. In addition to the scheduled quarterly assessments, when clinically feasible and safe, an intradialytic ultrasound examination may be performed for current IDH, suspected clinically significant fluid overload or suspected underestimated dry weight. The latter two indications correspond to a prescribed target weight suspected to be too high or too low, respectively. The same standardised acquisition protocol will be used when clinically feasible. Before ultrasound disclosure, the trigger indication and suspected direction of target-weight mismatch will be recorded; these variables will be described but will not define separate inferential subgroups. The number of IDH-affected sessions among the three preceding treatments will be derived from available dialysis records. The time from dialysis initiation to scan completion, prescribed session duration, cumulative ultrafiltration and current ultrafiltration rate, blood pressure at or immediately before imaging, and the temporal relation between scan completion and the first same-session IDH will be recorded or abstracted when available. Because IDH occurs throughout dialysis and the associations of selected haemodynamic variables with imminent IDH vary with time into treatment (19), scan completion will define the analysis landmark. Planned dialysis time remaining at scan completion will be derived as prescribed session duration in minutes minus elapsed time from dialysis initiation to scan completion. Eligibility for the separate post-scan association analysis requires an interpretable scan completed while the patient is clinically stable, before the first same-session IDH and not in response to evolving current-session haemodynamic instability. These scans will be followed from scan completion until the first subsequent IDH or session end. Scans obtained during or after IDH or during evolving haemodynamic instability will be used only to describe the peri-IDH haemodynamic phenotype.

Clinician questionnaire substudy (Objective 3). For each scheduled profiled session, the treating physician in charge will complete a brief structured questionnaire before disclosure of the study ultrasound findings. This pre-disclosure section will record the physician’s intended management for that session (for example ultrafiltration target, intended dry-weight adjustment, perceived congestion or underfilling profile, anticipated risk of intradialytic hypotension, and any planned adaptation of the dialysis prescription). These haemodynamic and volume-profile categories represent subjective clinical impressions and will not be interpreted as validated measurements of volume status. To prevent contamination of the primary IDH outcome, disclosure of the ultrasound findings will occur only after the dialysis session is complete and all intradialytic outcome data (blood pressure recordings, IDH events, nursing interventions, session completion status) have been recorded, except when findings meet the emergency disclosure criteria defined below. Once outcome data are secured, a second post-disclosure section will capture whether the findings provided clinically relevant new information, increased confidence in the prescription, and prompted a change in management. When the physician indicates a change, the corresponding modification is entered immediately in the routine dialysis prescription program. A positive post-disclosure response therefore documents an implemented prescription change after the index session rather than a hypothetical intention; it does not establish that the change improves clinical outcomes.

When feasible, a similar questionnaire may also be completed during event-triggered sessions. A reported change will likewise correspond to a modification entered in the routine dialysis prescription program. These responses will be reported in a prespecified descriptive subanalysis and will not be pooled with the scheduled-session Objective 3 analysis.

Observational design. This study remains observational. No study-mandated therapeutic algorithm will be applied, and the study team will not recommend or require any change in dialysis prescription on the basis of ultrasound findings. The questionnaire and disclosure procedure document the treating physician’s interpretation and any clinician-directed prescription change made after disclosure; they do not direct care. Any modification entered in the dialysis prescription program will remain entirely at the discretion of the treating physician as part of usual care. Standard care will otherwise proceed unchanged.

### Participants

Inclusion criteria are (1): adults aged ≥18 years receiving thrice-weekly maintenance haemodialysis for end-stage renal disease (2); receiving treatment at the study centre; and (3) able and willing to provide informed consent. Patients with a broad range of volume status and comorbidity profiles, including heart failure and diabetes, will be eligible provided that the other criteria are met.

Exclusion criteria are intentionally limited because of the observational design. Individuals will be excluded if they cannot safely undergo the ultrasound procedures or follow the protocol, for example because of an open chest wound or severe skin infection at probe sites, or because they lack capacity to consent and no legal representative is available. Persistent arrhythmia and valvular disease are not exclusion criteria, to preserve clinical representativeness. Rhythm and relevant valvular pathology at the time of scanning will be recorded; measurements that are not applicable or interpretable will be flagged accordingly. These participants will remain in the cohort, with prespecified stratified and sensitivity analyses where relevant. Pregnant individuals and prisoners or other persons unable to consent freely will be excluded. Patients receiving peritoneal dialysis are not eligible. If a participant receives a kidney transplant or transfers care during follow-up, active participation will end and data collected to that point will be retained for analysis.

We will recruit patients by approaching them during their routine dialysis sessions; information sheets and consent forms will be provided, and ample opportunity given to ask questions. Written informed consent will be obtained from all participants prior to any study-specific procedures.

### Schedule of assessments

The participant timeline and schedule of study procedures are shown in **Table 1**. Event-triggered POCUS examinations are intradialytic and will be performed only when clinically feasible and safe. Their time from dialysis initiation to scan completion and temporal relation to the first same-session IDH will be recorded. Routine disclosure of ultrasound findings occurs after dialysis-session outcome data have been recorded, except for prespecified emergency findings requiring immediate communication.

### Ultrasound assessment protocol

Each study ultrasound will be performed by a single trained operator, a nephrologist who completed a university-level echocardiography course at the Université Libre de Bruxelles in June 2023. All cardiac, lung and abdominal venous Doppler examinations will be performed in the dialysis unit using the same M5Sc-D XDclear matrix phased-array transducer on a GE Vivid S70N ultrasound system. Scheduled examinations will take place immediately before the assigned HD session (per prespecified allocation) and will be repeated at the end of the same session. The comprehensive assessment includes three domains:

#### Cardiac ultrasound

We will perform focused transthoracic echocardiography to evaluate cardiac function and haemodynamics. Key parameters will include left ventricular systolic function (visual estimate, with Simpson’s biplane ejection fraction when image quality permits), interventricular septal and posterior wall thickness, left ventricular outflow tract velocity-time integral (LVOT VTI), and estimated cardiac output. Additional markers of diastolic function will include mitral E and A velocities, E/A ratio, tissue Doppler e’ velocities, E/e’ ratio, left atrial volume, and pulmonary vein Doppler when feasible. These parameters will be recorded conditional on the predefined quality and interpretability criteria. Estimation of left ventricular filling pressure will follow a standardised algorithm based on American Society of Echocardiography recommendations for evaluation of left ventricular diastolic function (20). Right-heart assessment will include inferior vena cava (IVC) diameter and respiratory variation, basic right ventricular systolic function (tricuspid annular plane systolic excursion [TAPSE] and tricuspid S’), and the presence of pericardial effusion. When feasible, pulmonary artery systolic pressure will be estimated from peak tricuspid-regurgitation velocity and IVC-based estimated right atrial pressure, in accordance with current American Society of Echocardiography recommendations (21). In patients with arrhythmia, Doppler measurements will be averaged over ten consecutive beats.

#### Lung ultrasound

A standardised count-based eight-zone anterolateral protocol will be used. On each hemithorax, the anterior region (parasternal to anterior axillary line) and lateral region (anterior to posterior axillary line) will each be divided into superior and inferior zones. As a prespecified study convention, the superior and inferior zones will be separated by a horizontal line through the third intercostal space. Examinations will be performed with the patient fully supine using the same phased-array probe. Any clinically necessary deviation from the fully supine position will be recorded, and the same position will be reproduced for pre- and post-dialysis and serial examinations. A single focal point will be placed at the pleural line; tissue harmonics and spatial compound imaging will be disabled; and depth (typically approximately 15 cm and increased up to about 18 cm when required with the phased-array probe) and gain will be adjusted so that vertical artefacts can be followed to the bottom of the image, then kept constant whenever feasible. The probe will be held approximately perpendicular to the pleural surface. The entire accessible surface of each zone will be swept using an oblique intercostal view, and an eight-second cine loop will be stored (22, 23).

#### B-line quantification

A B-line will be defined as a discrete, laser-like vertical hyperechoic reverberation artefact arising from the pleural line, extending to the bottom of the screen without fading and moving synchronously with lung sliding when present; E-lines arising from subcutaneous tissue will not be counted. After review of the full cine loop, the frame with the maximum number of simultaneously visible B-lines will be selected. Counts from different frames or different points within the same zone will not be added, and the same artefact will not be counted repeatedly as it moves. Separated B-lines will be counted by distinct pleural origins: a single origin that fans or divides distally will count as one, whereas distinct origins that merge distally will be counted separately. When B-lines are confluent, the proportion of the image below the pleural line occupied by the confluent region will be estimated to the nearest 10%, with each 10% representing one B-line equivalent. Discrete B-lines outside the confluent region will be added without double counting; operationally, the zone count will be Z__j_ = min(10, D__j_ + C__j_), where D__j_ is the number of discrete B-lines outside confluent regions and C__j_ is the number of occupied 10% increments. The principal lung-ultrasound variable will be the sum of the eight zone counts (0–80). The number of positive zones (0–8), defined as at least three B-lines in a single frame or any confluent B-lines, will be a secondary lung-ultrasound variable. Zones wholly obscured by pleural effusion or with inadequate image quality will be coded as non-assessable rather than zero. The 0–80 total will be calculated only when all eight zones are assessable and will not be prorated. Pleural effusion will be recorded separately; consolidated lung will not be assigned an arbitrary B-line count, and any adjacent consolidation will be described in the lung-ultrasound comments. Pre- to post-dialysis change in total B-line count will be analysed as a marker of pulmonary decongestion (12, 22, 23).

#### Venous congestion ultrasound (modified VEXUS score)

Systemic venous congestion will be assessed using IVC diameter and respiratory variation, hepatic-vein Doppler and portal-vein Doppler. Hepatic-vein waveforms will be categorised as normal (S>D), mildly abnormal (S<D) or severely abnormal (systolic flow reversal). Portal-vein pulsatility will be categorised as normal, mildly abnormal or severely abnormal when the pulsatility fraction exceeds 50%. Intrarenal venous Doppler is omitted because its feasibility and interpretability are limited in patients with kidney failure, particularly in the presence of small or atrophic kidneys and altered renal perfusion. This approach mirrors the ACUVEX haemodialysis study, in which renal-vein Doppler was initially planned but subsequently omitted after renal venous flow proved mostly unrecordable and its validity and feasibility in end-stage kidney disease remained uncertain (13). The resulting IVC-hepatic-portal composite is therefore described as a prespecified modified VEXUS phenotype and is not presented as equivalent to the original VEXUS score or as a validated diagnostic or prognostic score in haemodialysis. ACUVEX provides population-specific support for feasibility and responsiveness to ultrafiltration but does not establish diagnostic or prognostic validity.

The analytic modified VEXUS grade will be derived directly from the recorded IVC, hepatic-vein and portal-vein component data. Grade 0 may be assigned when the IVC diameter is <2.0 cm. When the IVC diameter is ≥2.0 cm, both hepatic- and portal-vein Doppler waveforms must be interpretable: grade 1 denotes no severe Doppler abnormality, grade 2 one severe abnormality and grade 3 severe abnormalities in both veins. Technically inadequate or unavailable Doppler recordings will be coded as non-assessable rather than normal. If either waveform is unavailable or technically inadequate when the IVC diameter is ≥2.0 cm, the composite grade will be coded as non-assessable and will not be prorated. Available individual components will remain eligible for component-specific descriptive analyses. IVC collapsibility will be recorded separately but will not contribute to the modified grade. This prespecified algorithm is adapted from the published VEXUS framework (7). Measurements will be obtained supine before haemodialysis and repeated after haemodialysis. Event-triggered intradialytic measurements will use the same standardised supine position when clinically feasible; any deviation will be recorded.

All examinations will be stored for offline review. A prespecified random 20% sample from each ultrasound domain - cardiac, modified VEXUS and lung - will be independently re-analysed by a second experienced sonographer who did not perform the acquisition. The second reader will be either a practising cardiologist or a critical care physician holding EACVI Level 2 certification or higher. Pseudonymised cine loops and Doppler recordings will be reviewed without access to the first reader’s measurements or to clinical and IDH outcomes. Agreement will be evaluated using intraclass correlation coefficients and Bland-Altman analysis for continuous measurements and weighted Cohen’s κ for ordinal classifications.

### Quality assurance, interpretability and data completeness

For each examination, overall ultrasound quality and domain-specific quality (cardiac, venous congestion/VEXUS, and lung ultrasound) will be graded. The main limiting factors (e.g., poor acoustic windows, obesity/body habitus, chronic obstructive pulmonary disease (COPD)/emphysema, inability to position, arrhythmia, tachycardia, Doppler alignment, valvular disease, other) will also be recorded. Measures will be interpreted under prespecified, consensus-based quality criteria and according to international ultrasonography guidelines. All scans will be stored for later review.

Taxonomy of incomplete data. In multi-organ point-of-care ultrasound, it is anticipated that some measurements will be unavailable at certain sessions. The reasons for unavailability are not interchangeable and will be classified into four categories (1): not applicable - the measurement is structurally inapplicable to the patient (e.g., Doppler parameters requiring sinus rhythm in a patient with persistent atrial fibrillation; this is not “missing” and will not be imputed) (2); not interpretable - the measurement was attempted but the image or signal quality was insufficient for reliable interpretation despite adequate effort (e.g., poor transthoracic acoustic window due to body habitus, hyperinflation, or prior surgery; this represents a structural limitation and will not be imputed) (3); not obtained - the measurement could in principle have been obtained but was not attempted due to time constraints, operator availability, or logistical reasons (this is genuinely missing data and is a candidate for imputation under appropriate assumptions) (4); obtained but quality-limited - the measurement was obtained and recorded but flagged as marginal quality (these values will be included in the primary analysis with prespecified sensitivity analyses excluding them). The reason category will be recorded for every unavailable parameter at every session.

Because systematic inability to obtain a given domain is unlikely to be missing at random - patients with poor cardiac windows are more likely to have obesity, chronic lung disease, prior thoracic or cardiac surgery, or body habitus characteristics that may independently affect haemodynamic tolerance - partial-protocol data represent a potential source of informative missingness. To address this, several prespecified sensitivity analyses will be conducted (1): a complete-domain analysis restricted to sessions with fully interpretable cardiac, VEXUS, and lung ultrasound data (2); comparison of baseline characteristics, comorbidity burden, and outcome rates between patients with complete versus partial protocols, to characterise the direction and magnitude of any selection effect; and (3) domain-specific analyses in which each ultrasound component (cardiac, VEXUS, lung) is evaluated independently using all sessions for which that domain was interpretable, thereby maximising sample size per domain. The proportion, pattern, and reason category of incomplete data will be reported transparently.

### Outcomes and data collection

Outcomes are organised by analytic objective.

**Objective 1 - Descriptive characterisation**: Pre- and post-dialysis values of all ultrasound parameters listed in **Table 2**; within-session changes (pre-to-post); between-session trajectories across quarterly assessments; within-patient variability summaries.

**Table 2:** Prespecified ultrasound variables and clinical covariates.

Variable group and prespecified variables	Type	Domain/role	Timing
Left-ventricular diastolic measures: mitral E and A velocities; E-wave deceleration time; septal and lateral e’ velocities; left-atrial volume; pulmonary-vein Doppler when feasible	Continuous	Ultrasound outcomes	Scheduled pre + post; event-triggered when obtained
Left-ventricular systolic and haemodynamic measures: LV systolic function (4-level visual grade); LVOT VTI; heart rate; Simpson biplane LVEF when feasible	Continuous/ordinal	Ultrasound outcomes	Scheduled pre + post; event-triggered when obtained
Right-heart measures: TAPSE; tricuspid S’; estimated right-atrial pressure; estimated pulmonary-artery systolic pressure when feasible; pericardial effusion	Continuous/ordinal	Ultrasound outcomes	Scheduled pre + post; event-triggered when obtained
Modified VEXUS measures: IVC diameter; IVC collapsibility; hepatic-vein Doppler; portal-vein pulsatility; modified VEXUS grade	Continuous/ordinal	Ultrasound outcomes	Scheduled pre + post; event-triggered when obtained
Lung-ultrasound measures: total eight-zone B-line count (0–80); positive-zone count (0–8); pleural effusion; adjacent consolidation recorded descriptively	Counts/ordinal	Ultrasound outcomes	Scheduled pre + post; event-triggered when obtained
Session-level dialysis variables: rhythm at scan; ultrafiltration target and delivered volume; interdialytic weight gain; pre-dialysis systolic BP; session type; dialysate sodium, calcium and temperature; prescribed session duration; for triggered scans, indication and suspected direction, time from dialysis initiation to scan completion, cumulative ultrafiltration and current ultrafiltration rate, BP at scan, recent IDH history, temporal relation to the first same-session IDH, and derived planned dialysis time remaining	Continuous/categorical	Time-varying covariates	Per session; at event-triggered scan
Baseline demographic and dialysis characteristics: age; sex; dialysis vintage; vascular-access type; residual diuresis; kidney-failure aetiology; dialysis modality (HD/HDF)	Continuous/categorical	Baseline covariates	Baseline
Comorbidities: diabetes; hypertension; coronary-artery disease; heart failure and phenotype; COPD	Categorical	Confounders/effect modifiers	Baseline
Baseline structural cardiac variables: prior LVEF; interventricular-septal and posterior-wall thickness	Continuous	Baseline structural variables	Baseline
Medication variables: ACE inhibitor or angiotensin-receptor blocker; beta-blocker; diuretic use	Binary	Baseline/session covariates	Baseline
Laboratory variables: serum albumin; haemoglobin	Continuous	Baseline covariates	Routine clinical measurements
Derived ultrasound variables: E/A; septal and average E/e’; stroke volume; cardiac output; pulmonary-artery systolic pressure; left-atrial-volume index; pulmonary-vein S/D; modified VEXUS grade; diastolic-dysfunction grade	Derived continuous/ordinal	Reported derivatives; not entered with their component measures in the same model	Derived from corresponding assessment

ACE, angiotensin-converting enzyme; BP, blood pressure; COPD, chronic obstructive pulmonary disease; HD, haemodialysis; HDF, haemodiafiltration; IVC, inferior vena cava; LVEF, left-ventricular ejection fraction; LVOT VTI, left-ventricular outflow-tract velocity-time integral; TAPSE, tricuspid annular plane systolic excursion; VEXUS, venous excess ultrasound.

**Objective 2 - Ultrasound profile and intradialytic hypotension**: within-patient analyses (primary outcome). Two definitions of IDH will be applied to every profiled session and reported separately. Blood pressure is measured immediately before dialysis, every 30 minutes throughout treatment, at the end of dialysis and additionally whenever compatible symptoms occur or staff suspect haemodynamic instability. The immediately pre-dialysis measurement is used as the reference value, and the session nadir is the lowest systolic blood pressure obtained from either scheduled or symptom-triggered measurements. A primary IDH event requires a documented decrease in systolic blood pressure of at least 20 mmHg from the pre-dialysis reference value and either at least one prespecified compatible symptom or at least one acute nursing intervention undertaken specifically in response to the episode. Symptoms and interventions are recorded separately using prespecified case-report-form categories. Interventions include saline administration, ultrafiltration-rate reduction or cessation, repositioning, premature session termination and acute administration of an indicated vasoactive treatment. This clinically consequential definition was prespecified for Objective 2 because it permits real-time case ascertainment during scheduled and event-triggered sessions. The secondary definition is a nadir systolic blood pressure below 90 mmHg at any point during the session, or below 100 mmHg when the pre-dialysis systolic blood pressure is at least 160 mmHg. This nadir-based definition demonstrated the most consistent association with mortality in the analysis by Flythe et al. (24) and will be applied using all scheduled and symptom-triggered blood-pressure measurements. Sensitivity analyses will examine whether findings differ according to the IDH definition. The primary Objective 2 analysis uses only scheduled profiled sessions. Event-triggered examinations will not contribute cases to the primary analysis or to another scheduled-session model. Eligible event-triggered scans completed while the patient is clinically stable and before the first same-session IDH will enter the separate post-scan association analysis of subsequent IDH. Scans obtained during or after IDH or during evolving haemodynamic instability will be described only. Additional intradialytic outcomes reported include premature session termination, reduced delivered dialysis dose because of intolerance, ultrafiltration reduction or interruption and saline administration.

**Objective 3 - Clinician-directed prescription changes after ultrasound disclosure (exploratory)**: For each scheduled profiled session, whether post-session disclosure of the ultrasound findings results in a change entered in the dialysis prescription program, analysed as a binary variable (change vs no change) and descriptively by domain of change. The physician’s rating of clinical relevance and confidence in the management decision will also be captured. Pre-disclosure haemodynamic and volume-profile classifications will be treated as subjective clinician impressions and will not be used to estimate diagnostic accuracy or to classify patients as objectively overloaded, euvolaemic or underfilled.

**Objective 4 - Longitudinal clinical outcomes (exploratory)**: All-cause hospitalisations, particularly those related to cardiovascular causes or fluid overload; cardiovascular events (acute coronary syndromes, heart failure exacerbations, strokes, arrhythmias); and all-cause mortality. These outcomes will be tracked from medical records over the study duration (anticipated 12 months). Given the expected small number of events (approximately 9–12 deaths), all Objective 4 analyses are explicitly exploratory and hypothesis-generating.

Session-level data. For each scheduled ultrasound time-point, routinely available session-level data will be recorded when captured in the case report form, including blood pressure, heart rate, pre- and post-dialysis weight, ultrafiltration volume, prescribed dialysis-session characteristics and IDH events or interventions documented by dialysis staff. Blood pressure is recorded immediately before dialysis, every 30 minutes during treatment, at the end of treatment and additionally when symptoms occur or staff suspect haemodynamic instability; the session nadir is the lowest scheduled or clinically triggered measurement. Session type (post-long interdialytic interval vs mid-/late-week) will be recorded for each profiled visit. For every event-triggered scan, the clinical indication, suspected direction of target-weight mismatch, time from dialysis initiation to scan completion, prescribed session duration, cumulative ultrafiltration delivered, current ultrafiltration rate, blood pressure at or immediately before imaging, temporal relation to the first same-session IDH episode and recent IDH burden will be recorded or abstracted from routine treatment records when available. Planned dialysis time remaining at scan completion will be derived as prescribed session duration in minutes minus elapsed time to scan completion.

### Clinician questionnaire and disclosure procedure

For each scheduled ultrasound time-point, the treating physician responsible for the session will initially remain blinded to the study ultrasound findings. Before the session begins, the physician will complete a short questionnaire recording the intended management for that session. The questionnaire will record the planned ultrafiltration target, intended change in dry weight, the physician’s assessment of the patient’s predominant haemodynamic or volume profile, anticipated risk of intradialytic hypotension, and any intended adaptation of the dialysis prescription. The haemodynamic and volume-profile categories are deliberately coarse, subjective clinical impressions; no quantitative threshold or objective reference standard is applied, and they will not be interpreted as validated classifications of true volume status.

The questionnaire will not collect the physician’s name, e-mail address, initials, stamp number or any other nominative physician identifier. Responses will be analysed at session/patient level only and will not be reported by individual physician.

After the dialysis session is complete and all intradialytic outcome data have been recorded (including blood pressure readings, IDH events, nursing interventions, saline administration, ultrafiltration modifications, and session completion status), the ultrasound findings from the study examination will be disclosed to the physician. A second section of the questionnaire will then record whether the findings were considered clinically relevant, whether they prompted a change in management, which specific aspects were modified (e.g., ultrafiltration target, dry-weight strategy, session duration, sodium or temperature prescription, additional investigations), and whether the findings increased confidence in the prescription. When a change is indicated, the corresponding modification is entered immediately in the routine dialysis prescription program. This provides evidence of an implemented prescription change after disclosure, while the post-session timing ensures that the primary IDH outcome remains uncontaminated by knowledge of the ultrasound findings.

**Exception: emergency disclosure**. The post-session disclosure rule is overridden when the pre-dialysis ultrasound reveals prespecified findings of immediate clinical concern, as defined in the Ethical and Safety Considerations section below.

### Statistical analysis plan

The analyses for each objective are described below. All analyses will be performed in R (version ≥4.3). The statistical analysis plan follows published recommendations for statistical analysis plans in observational studies (25) and was finalised before any study data were examined. All planned analyses, including their designation as primary, secondary or exploratory, were decided *a priori*; any *post-hoc* analyses will be clearly labelled as such in the final report. Reporting of the completed cohort will follow the STROBE statement and its explanation and elaboration (26, 27).

Analysis populations. Three analysis populations are defined. The full analysis set includes all enrolled participants who undergo at least one scheduled ultrasound assessment. The per-protocol set includes participants who complete all four quarterly assessments with interpretable data in at least one ultrasound domain. The event-triggered analysis set includes participants with at least one interpretable event-triggered examination. Within the event-triggered analysis set, eligibility for the separate post-scan association model will be determined at scan level: only scans completed while the patient is clinically stable, before the first same-session IDH and not in response to evolving current-session haemodynamic instability will contribute. Scans obtained during or after IDH or during evolving instability will be retained for descriptive analyses. Trigger indication will not define separate analysis populations or inferential subgroups. Objective 1 analyses will use the full analysis set. The primary Objective 2 case-crossover is defined within the full analysis set, but conditional logistic regression derives outcome information only from participants contributing at least one scheduled IDH session and at least one scheduled non-IDH session. For a given ultrasound parameter, contribution also requires within-patient variation in that parameter. Prespecified sensitivity analyses will include repetition on the per-protocol set to assess the impact of incomplete follow-up, on the complete-domain set (sessions with all three ultrasound domains interpretable) to evaluate partial-protocol bias, and under the alternative IDH definitions described above. The analysis population used for each sensitivity analysis will be reported explicitly.

Multiplicity and analytic hierarchy. Objective 1 is descriptive and will be reported using estimates and confidence intervals. The scheduled-session case-crossover is the primary prespecified analysis for Objective 2 but is exploratory and non-confirmatory; its primary status denotes its position in the analytic hierarchy. Objective 2 analyses will emphasise effect estimates and 95% confidence intervals rather than decisions based on a fixed type I error threshold. Exact p-values may be reported descriptively without dichotomisation. Where multiple related secondary tests are performed, false-discovery-rate-adjusted values may be provided as an aid to interpretation but will not confer confirmatory status. Objectives 3 and 4 and all subgroup analyses remain exploratory and hypothesis-generating. Any subsequent amendments to this prespecified analysis plan will be dated, documented and justified.

Confounder adjustment strategy. In the patient-stratified scheduled-session case-crossover analysis, time-invariant participant characteristics (age, sex, diabetes status, dialysis vintage and vascular access type) are controlled by design and will not be estimated as covariates. Candidate time-varying confounders are prespecified as pre-dialysis systolic blood pressure, interdialytic weight gain and prescribed ultrafiltration volume. For the separate event-triggered post-scan association analysis, planned dialysis time remaining at scan completion will be included in each model. This variable will be derived as 60 multiplied by prescribed session duration in hours, minus the number of minutes from dialysis initiation to scan completion. Scan-time systolic blood pressure, cumulative ultrafiltration and recent IDH burden will be described and may be added individually in sensitivity models if supported by the number of subsequent IDH events and model stability. Trigger indication will not define inferential subgroups. In scheduled-session mixed-effects and exploratory longitudinal models, the same baseline and time-varying variables may be included when clinically justified and when the number of events permits stable estimation. Confounder selection is based on prespecified clinical causal reasoning rather than observed imbalance in descriptive baseline tables. Adjusted case-crossover analyses will be undertaken only if supported by the number of outcome-informative patient strata, within-patient variability of the relevant ultrasound parameters and model stability; otherwise, analyses will be univariable or minimally adjusted.

**Prespecified variables**. The ultrasound outcomes, clinical covariates and derived variables used in the analyses are listed in **Table 2**.

#### Objective 1: descriptive characterisation

**Stage 1a - IDH patterns**. At the session level, the overall IDH rate will be reported as n(IDH sessions)/N(total sessions) with a 95% confidence interval accounting for within-patient clustering (from a generalised linear mixed model intercept-only model). At the patient level, each patient’s IDH proportion across their sessions will be computed and reported as mean ± SD and median (IQR). Patients will be classified as having “frequent IDH” if ≥30% of their sessions are affected, for descriptive purposes. A histogram of patient-level IDH proportions will be presented. The ICC for IDH across sessions within patients will be reported as a descriptive measure of outcome clustering; it does not replace reporting the number of outcome-informative patient strata for the case-crossover analysis.

Ultrasound parameters will be summarised according to their distributional properties: continuous variables as mean ± SD or median (IQR), according to distributional shape assessed by graphical inspection and Shapiro-Wilk testing, and ordinal variables (e.g., VEXUS grade) as frequencies and percentages by category. Baseline characteristics will be presented descriptively according to IDH-frequency group (frequent vs infrequent). Between-group differences will be described using standardised mean differences rather than hypothesis tests; an absolute standardised mean difference >0.1 will be flagged as potentially meaningful but will not determine which confounders are adjusted for, because the adjustment strategy is prespecified from clinical causal reasoning.

**Stage 1b - Within-session pre- versus post-dialysis comparisons**. Univariate paired comparisons will be performed for each ultrasound outcome variable listed in **Table 2**. The choice between parametric and non-parametric tests will depend on the distribution of within-session differences assessed by graphical inspection and Shapiro-Wilk testing; ordinal variables will use the Wilcoxon signed-rank test. For dichotomised thresholds, McNemar’s test will be applied. Ultrasound outcomes will also be analysed jointly using multivariate mixed-effects models with outcome-specific submodels, appropriate distributions and estimated residual correlations between outcomes. Session-level and patient-level covariates from **Table 2** will be included in models of pre-to-post change. This approach is preferred over repeated-measures MANOVA, which requires multivariate normality and complete data and cannot accommodate ordinal outcomes. Exploratory visual analyses will include (1): spaghetti plots of individual pre-to-post trajectories coloured by clinical subgroup (2); forest plots of subgroup-specific pre-post effect estimates with 95% CIs (3); interaction plots showing predicted mean change as a function of ultrafiltration volume stratified by comorbidity; and (4) stratified correlation heatmaps of pre-to-post change scores.

**Stage 1c - Between-session variability**. Within-patient trajectories of each ultrasound parameter (**Table 2**) across quarterly assessments will be modelled using linear mixed models with random intercepts and, where supported by the data, random slopes per patient. The intraclass correlation coefficient will be reported for relative reliability; modified Bland-Altman analyses using within-subject standard deviations will assess absolute agreement. To investigate whether within-patient variability of ultrasound parameters is itself associated with IDH frequency, mixed-effects location-scale models (28) will be explored as a prespecified secondary analysis if data completeness and model convergence permit. These models jointly describe the mean response and within-person variance as functions of covariates, allowing each patient an intrinsic variability level. In practical terms, the analysis asks whether patients whose ultrasound parameters fluctuate more from session to session also experience more IDH, beyond differences in their average ultrasound profile. The variance submodel will include session-level covariates from **Table 2** as potential explanatory variables. With only four quarterly observations per patient, patient-specific variance estimates will be imprecise and will therefore be interpreted cautiously.

**Stage 1d - Dimension reduction**. Multilevel principal component analysis (PCA) will be applied to the ultrasound outcome variables (**Table 2**) to decompose the total covariance into between-patient and within-patient components. Patient-level means and within-patient standard deviations of PC scores will be computed as summary measures of individual ultrasound profile stability. Uniform Manifold Approximation and Projection (UMAP) will be used for exploratory visualisation of patient clusters but will not replace PCA in formal models.

**Event-triggered intradialytic scans**. Event-triggered examinations will be summarised by clinical indication and temporal relation to the first same-session IDH: stable before the first IDH with subsequent IDH, stable before the first IDH without subsequent IDH, during or in response to evolving IDH, or after IDH. These categories are used to define temporal eligibility and descriptive presentation, not indication-based inferential subgroups. The number of scans and contributing patients, time from dialysis initiation to scan completion, planned dialysis time remaining, scan-time blood pressure, cumulative ultrafiltration, current ultrafiltration rate, recent IDH burden and ultrasound findings will be summarised. Only stable scans completed before the first same-session IDH will enter the separate post-scan association model described below. Scans obtained during or after IDH or during evolving haemodynamic instability will contribute only to contemporaneous or post-event phenotyping. Event-triggered scans will not be pooled with scheduled pre- or post-dialysis measurements. Because event-triggering reflects clinical suspicion and may be informed by recent IDH, the findings describe an indication-enriched sample.

#### Objective 2: ultrasound profile and intradialytic hypotension

**Primary analysis: scheduled-session case-crossover**. The primary prespecified Objective 2 analysis will use conditional logistic regression stratified by patient to compare pre-dialysis ultrasound parameters at scheduled profiled sessions where IDH occurs with the same patient’s scheduled profiled sessions where IDH does not occur. Restriction to scheduled pre-dialysis scans ensures that ultrasound measurements precede dialysis and preserves the predictive interpretation of the estimand. This primary analysis is exploratory and non-confirmatory. Conditional logistic regression derives information only from participants contributing at least one scheduled IDH session and at least one scheduled non-IDH session. Under the simplifying assumptions of four independent sessions and a constant 10% event probability, approximately 21 of 60 participants would be outcome-informative; allowing for 10% missing sessions reduces this estimate to approximately 19. These estimates may be optimistic because IDH may cluster within susceptible participants and because an ultrasound parameter must vary within a participant to contribute information about its association with IDH. Adjusted analyses will be undertaken only if the number of outcome-informative patient strata and the within-patient variation in the ultrasound parameter permit stable estimation; otherwise, results will be restricted to univariable or minimally adjusted associations. The numbers of IDH events, outcome-informative patient strata, case and control sessions, and the within-patient variability of the analysed ultrasound parameters will be reported. Null findings will be interpreted as inconclusive rather than as evidence of no association.

**Secondary analysis: event-triggered intradialytic post-scan association model**. Scan completion will define the index time. Model-eligible observations are interpretable event-triggered scans completed while the patient is clinically stable, before the first qualifying same-session IDH and not in response to evolving current-session haemodynamic instability. Each eligible scan will be followed from scan completion until session end, and the binary outcome will be the occurrence of at least one subsequent IDH. Scans without a subsequent IDH will constitute the post-scan non-event comparison observations. A generalised linear mixed-effects model with a logit link and a patient-level random intercept will be fitted separately for each prespecified ultrasound parameter. Each model will include planned dialysis time remaining at scan completion, derived as 60 multiplied by prescribed session duration in hours, minus the number of minutes from dialysis initiation to scan completion. Scan-time systolic blood pressure, cumulative ultrafiltration and recent IDH burden may be added individually in sensitivity analyses if the number of subsequent IDH events permits stable estimation; they will not all be entered routinely. Trigger indication will be reported descriptively and will not define separate inferential models. Scans obtained during or after IDH or during evolving instability will not enter this model and will be used only to describe the peri-IDH haemodynamic phenotype. The numbers of eligible scans, subsequent IDH events, post-scan non-event observations, contributing patients and repeat observations per patient will be reported with effect estimates and 95% confidence intervals. If the numbers of eligible scans, subsequent IDH events or contributing patients are insufficient for stable mixed-effects estimation, the triggered-scan findings will remain descriptive. These results will not be interpreted as population-level prediction, diagnostic accuracy or causal effects. The direction and magnitude of associations will be compared descriptively with the scheduled-session results; no formal equivalence claim will be made.

**Scheduled-session prognostic modelling (secondary)**. A generalised linear mixed model with a logit link and a random intercept per patient will estimate the association between pre-dialysis ultrasound parameters measured during scheduled profiled sessions and same-session IDH. This analysis is restricted to scheduled pre-dialysis scans so that it retains a prognostic interpretation. Candidate predictors will be restricted to a small prespecified set, with final model complexity determined by the observed number of IDH events, participating patients and model stability. Penalised estimation (ridge or elastic net) may be used to reduce overfitting. If the data do not support a stable multivariable model, results will be limited to univariable or minimally adjusted associations and no prognostic-model-development claim will be made. Where a multivariable model is estimable, confidence intervals will be obtained by resampling whole patients rather than individual sessions. If derived profile scores from Stage 1d are incorporated, dimension reduction will be refitted within each training fold and held-out patients will be projected into the training-derived component space to avoid information leakage.

Internal validation of the exploratory scheduled-session prognostic model. If an adequately stable multivariable model is developed, internal validation will use leave-one-patient-out cross-validation. Performance measures will include a cluster-aware area under the receiver-operating-characteristic curve with bootstrap confidence intervals, calibration slope and calibration plots. Reporting will follow applicable TRIPOD+AI and TRIPOD-Cluster recommendations (29, 30). The exploratory nature of the analysis and the limited events-per-variable ratio will be stated explicitly. No external validation is planned. The purpose is to identify associations for evaluation in a larger confirmatory cohort.

#### Objective 3: clinician-directed prescription changes after ultrasound disclosure

The proportion of scheduled profiled sessions in which ultrasound disclosure results in an implemented prescription change, defined by entry of the corresponding modification in the routine dialysis prescription program, will be reported with a 95% confidence interval accounting for repeated observations within patients. Because no nominative physician identifier is collected, clinician-level clustering cannot be estimated. Changes will be tabulated by domain (ultrafiltration target, dry-weight strategy, dialysis-session adaptation, antihypertensive management, need for an additional session and further diagnostic work-up). Pre-disclosure haemodynamic and volume-profile categories and post-disclosure ultrasound interpretations will be summarised descriptively and will not be used for diagnostic-accuracy analyses. The physician’s rating of clinical relevance and change in confidence will also be summarised descriptively. No multivariable modelling of prescription change after ultrasound disclosure is planned. This substudy documents prescribing behaviour after ultrasound disclosure but does not determine whether the resulting changes improve clinical outcomes.

#### Objective 4: longitudinal clinical outcomes

For time-to-event outcomes (hospitalisations, cardiovascular events and mortality), joint models for longitudinal and survival data are planned, conditional on sufficient event counts and model convergence, to relate ultrasound trajectories to event hazards. Models will be implemented using JMbayes2 (31). Given the expected small number of deaths (approximately 9–12 over 12 months), mortality analyses are explicitly exploratory and hypothesis-generating and will be restricted to very simple models. A prespecified two-stage analysis will also be performed: mixed models will first characterise each patient’s ultrasound trajectory, and the resulting summary measures will then be entered into Cox proportional-hazards models. If the joint models cannot be fitted, the two-stage analysis will instead be used to describe associations between ultrasound trajectories and clinical outcomes. Competing-risk analyses for kidney transplantation and recurrent-event analyses for hospitalisations will be undertaken only if the observed event counts permit stable estimation.

### Missing data

The primary analysis will use all available data under a missing-at-random assumption, with domain-specific analyses as described in the quality assurance section above. Only data classified as “not obtained” (category 3 in the taxonomy above) are candidates for imputation. Data classified as “not applicable” or “not interpretable” (categories 1 and 2) represent structural limitations and will not be imputed; these will be handled through domain-specific analyses that use all sessions for which each domain was interpretable. For multivariable models requiring multiple ultrasound domains simultaneously, multiple imputation by chained equations will be performed using at least 20 imputed datasets pooled with Rubin’s rules, restricted to parameters with a “not obtained” reason for missingness. A sensitivity analysis under plausible missing-not-at-random assumptions will use a δ-adjustment approach only for data classified as “not obtained” (category 3) where informative missingness is clinically plausible. Complete-case analysis will also be reported. If more than 40% of observations on any domain are unavailable, that domain will be treated as secondary or exploratory for multivariable analyses.

### Sample size

This is an exploratory single-centre prospective observational cohort study. The target of approximately 60 participants is primarily feasibility-based, reflecting achievable recruitment and longitudinal follow-up within the haemodialysis unit. Four scheduled assessments per participant would provide a maximum of 240 profiled sessions; after allowing approximately 10% for missed visits, incomplete assessments or withdrawal, approximately 216 scheduled sessions are expected to remain available for analysis.

The sample-size rationale is based on feasibility, anticipated event counts and the precision of descriptive scheduled-session estimates rather than on formal power for a specified conditional odds ratio (32). Reported session-level IDH frequencies vary substantially according to the definition used. A systematic review and meta-analysis reported prevalence estimates of approximately 10.1% using the EBPG definition and 11.6% using a nadir systolic blood pressure below 90 mmHg, while emphasising substantial heterogeneity across studies and definitions (33). A session-level frequency of 10% was therefore used as a planning assumption.

Using a planning assumption of an overall scheduled-session intradialytic hypotension rate of 10%, 240 scheduled sessions would yield approximately 24 events and 216 sessions approximately 22 events. Because repeated sessions from the same patient are not statistically independent, a simple design-effect correction was applied for within-patient clustering (34). Under DE = 1 + (m - 1) × ICC, with m = 4 scheduled sessions per patient and a plausible within-patient intraclass correlation range of 0.10 to 0.20, the design effect is 1.3 to 1.6. The corresponding effective sample size is approximately 150 to 185 for 240 sessions and 135 to 166 for 216 sessions. Across the assumed ICC range, the approximate 95% confidence interval half-width around a 10% IDH proportion is 4.3% to 4.8% for 240 sessions and 4.6% to 5.1% for 216 sessions. These effective-sample-size and confidence-interval calculations apply only to estimation of the descriptive scheduled-session IDH proportion and do not quantify the precision or statistical power of the conditional odds ratio in the case-crossover analysis.

**Sample-size implications by analytic objective**. For Objective 1, the sample is intended to provide descriptive estimates of ultrasound distributions, data completeness, within-session change and between-session variability. For Objective 2, the scheduled-session case-crossover derives information from outcome-discordant within-patient strata rather than from the total number of sessions. No formal power claim is therefore made for a specified conditional odds ratio or fixed multivariable model. Under the 10% planning assumption, approximately 24 scheduled-session IDH events are expected with complete follow-up and approximately 22 after allowing for 10% missing or incomplete visits. Under the simplifying assumptions of independent sessions and a constant event probability, approximately 21 participants would contribute both a scheduled IDH and a scheduled non-IDH session with complete follow-up, decreasing to approximately 19 after allowing for missing sessions. These estimates may be optimistic because IDH may cluster within susceptible participants and individual ultrasound parameters must also vary within patients. The scheduled-session case-crossover remains the primary prespecified Objective 2 analysis but is exploratory and non-confirmatory. Adjusted analyses will be undertaken only if the number of outcome-informative patient strata and the within-patient variation in the ultrasound parameter permit stable estimation. Event-triggered examinations will not increase the primary scheduled-session case count. The feasibility of the separate event-triggered post-scan association model will depend on the number of stable scans completed before the first same-session IDH, the number of subsequent IDH events and the number of patients contributing scans. No formal power claim is made for this exploratory analysis; if the numbers of eligible scans, subsequent IDH events or contributing patients are insufficient for stable mixed-effects estimation, results will remain descriptive. For Objective 3, up to approximately 240 scheduled questionnaires are expected. For Objective 4, the anticipated 9–12 deaths permit only simple exploratory analyses and may preclude stable joint modelling.

Because the prevalence of ultrasound-defined haemodynamic profiles is not known *a priori* and is not controlled by the investigators, a formal sample-size calculation based on comparing intradialytic hypotension rates between predefined profile groups would require speculative assumptions about both the distribution of profiles and the magnitude of between-profile differences. The analytic ambition of each objective has been calibrated to the expected information content of this dataset.

### Data management

Study data will be entered into a secure institutional database. Participants will be identified in the analysis dataset by a study ID only. Direct identifiers, including the patient’s name, initials, hospital number/ID Erasme, national registry number and full date of birth, will not be included in exported analysis datasets. A separate code-identification list linking the study ID to participant identity will be maintained locally, stored separately in a secure access-restricted file, and accessible only to authorised study investigators designated in the ethics file. Age at inclusion will be used in the analysis dataset instead of full date of birth. Digital ultrasound images/clips will be pseudonymised before study archiving and stored on secure hospital servers for offline analysis and quality control. The pseudonymised data and essential study documents will be retained for 20 years after study completion, unless a longer legal or institutional requirement applies. For independent offline remeasurement, a prespecified random 20% sample from each ultrasound domain - cardiac, modified VEXUS and lung - will be reviewed by the second experienced sonographer described above, blinded to the first reader’s measurements and to clinical and IDH outcomes.

## Discussion

This protocol describes a structured, multi-organ ultrasound approach to haemodynamic profiling in maintenance haemodialysis. The design deliberately separates descriptive characterisation, within-patient IDH analyses, clinician-perceived usefulness and clinician-directed prescription changes after ultrasound disclosure, and exploratory longitudinal outcomes, thereby reducing the risk that a broad observational dataset is interpreted through *post-hoc* outcome selection.

A key strength is the embedded case-crossover analysis, which uses each participant as their own control and therefore controls by design for time-invariant between-patient confounding. Restricting the primary analysis to scheduled pre-dialysis scans preserves the predictive interpretation of the main estimand. The scheduled-session case-crossover remains primary because it directly addresses the central within-patient question while preserving temporal ordering, but it is explicitly exploratory and non-confirmatory. The event-triggered substudy is kept analytically separate: stable scans completed before the first same-session IDH may enter the exploratory post-scan association model of subsequent IDH, whereas scans obtained during or after IDH or during evolving haemodynamic instability will be used only to describe the peri-IDH haemodynamic phenotype.

The study also addresses important practical limitations of point-of-care ultrasound in haemodialysis. Imaging quality, rhythm, valvular disease and incomplete domain acquisition are explicitly captured, and the missing-data taxonomy distinguishes structural non-interpretability from genuinely missed measurements. The single-centre design and modest sample size limit external validity and preclude definitive prognostic-model development, but the study is designed to estimate data completeness, ultrasound variability, within-session change and preliminary associations that can inform a larger multicentre cohort or future interventional trial.

Several limitations are prespecified. The observational design does not test whether an ultrasound-guided treatment strategy improves clinical outcomes. The questionnaire’s haemodynamic and volume-profile categories are subjective clinical impressions without a quantitative threshold or objective reference standard. Positive post-disclosure responses correspond to prescription changes entered in the dialysis prescription program and therefore document implemented management changes; however, the descriptive substudy cannot establish whether those changes are appropriate or beneficial. The primary scheduled-session case-crossover may be constrained by approximately 22 IDH events and fewer outcome-informative patient strata. Estimates may therefore be imprecise, and null findings will be considered inconclusive. Event-triggered scans are selected by clinical indication and may be prompted by recent IDH. Even among model-eligible stable scans, scan timing and planned observation time vary, and unmeasured clinical factors may influence both scanning and subsequent IDH. The separate post-scan association model therefore estimates an exploratory association within an indication-enriched sample and cannot be interpreted as population-level prediction, diagnostic accuracy or a causal effect. Ultrasound phenotypes obtained during or after IDH may reflect acute treatment as well as the underlying physiology and will therefore remain descriptive. The modified IVC-hepatic-portal VEXUS phenotype is not interchangeable with the original VEXUS score, and its diagnostic and prognostic validity in maintenance haemodialysis has not been established. Use of a single trained operator standardises image acquisition. The independent second-reader assessment evaluates inter-reader interpretation and measurement agreement but does not assess variability arising from image acquisition by different operators. This absence of an inter-operator acquisition assessment is an acknowledged limitation. Finally, exploratory longitudinal analyses may be constrained by low event counts and model convergence.
