# Supplementary material for: Ultrasound-guided haemodynamic profiling in haemodialysis patients: a single-centre prospective observational study protocol
Source: Front Nephrol. 2026 Oct 20;6:1939088. doi: 10.3389/fneph.2026.1939088 (PMC13727544; doi:10.3389/fneph.2026.1939088)
Supplement: Supplementary file 1 [file Table1.docx]

**Supplementary File 3. Clinician questionnaire**

Publication version. Tick one response unless "select all that apply" is stated. Complete Part A before reviewing the study ultrasound findings. Complete Part B only after the dialysis session is complete, all intradialytic outcome data have been recorded and the study ultrasound findings have been disclosed. The questionnaire records hypothetical clinician-reported management impact and does not direct care during the index session. Nominative physician identifiers are omitted from this publication version.

**Administrative fields**

| **Participant study ID** | ____________________________ |
| --- | --- |
| **Visit / session** | ☐ Baseline ☐ Month 3 ☐ Month 6 ☐ Month 9 ☐ Event-triggered ☐ Other: __________ |
| **Date** | ____ / ____ / ______ (DD/MM/YYYY) |

**Part A - before reviewing the study ultrasound results**

**1. What is your planned ultrafiltration target for this session?**

________________ mL

**2. Before reviewing the study ultrasound results, were you planning to modify the patient's estimated dry weight?**

| ☐ No | ☐ Yes, decrease it |
| --- | --- |
| ☐ Yes, increase it |  |

**3. How would you classify the patient's current volume/haemodynamic profile?**

| ☐ At risk of hypovolaemia | ☐ Euvolaemic |
| --- | --- |
| ☐ At risk of fluid overload | ☐ Mixed profile |

**4. What is your assessment of the risk of intradialytic hypotension during this session?**

| ☐ Very low | ☐ Low |
| --- | --- |
| ☐ Moderate | ☐ High |
| ☐ Very high |  |

**5. Were you planning any specific adaptation of the dialysis prescription?**

*Select all that apply.*

| ☐ No | ☐ Reduce ultrafiltration |
| --- | --- |
| ☐ Increase ultrafiltration | ☐ Extend the session |
| ☐ Reduce blood-flow rate | ☐ Change dialysate temperature |
| ☐ Change sodium prescription/profile | ☐ Other: __________________________ |

**6. How comfortable are you interpreting cardiac ultrasound findings?**

*0 = not at all comfortable; 10 = fully comfortable.*

| **Score** | **0** | **1** | **2** | **3** | **4** | **5** | **6** | **7** | **8** | **9** | **10** |
| --- | --- | --- | --- | --- | --- | --- | --- | --- | --- | --- | --- |
| Tick | ☐ | ☐ | ☐ | ☐ | ☐ | ☐ | ☐ | ☐ | ☐ | ☐ | ☐ |

**7. How comfortable are you interpreting lung ultrasound findings?**

*0 = not at all comfortable; 10 = fully comfortable.*

| **Score** | **0** | **1** | **2** | **3** | **4** | **5** | **6** | **7** | **8** | **9** | **10** |
| --- | --- | --- | --- | --- | --- | --- | --- | --- | --- | --- | --- |
| Tick | ☐ | ☐ | ☐ | ☐ | ☐ | ☐ | ☐ | ☐ | ☐ | ☐ | ☐ |

**8. How comfortable are you interpreting VEXUS findings?**

*0 = not at all comfortable; 10 = fully comfortable.*

| **Score** | **0** | **1** | **2** | **3** | **4** | **5** | **6** | **7** | **8** | **9** | **10** |
| --- | --- | --- | --- | --- | --- | --- | --- | --- | --- | --- | --- |
| Tick | ☐ | ☐ | ☐ | ☐ | ☐ | ☐ | ☐ | ☐ | ☐ | ☐ | ☐ |

**Part B - after delayed disclosure of the study ultrasound results**

**9. Did the ultrasound results provide clinically relevant information?**

| ☐ No, no change in management is needed | ☐ Yes, they support my clinical impression that management should be changed |
| --- | --- |
| ☐ Yes, they alert me to a need to change management |  |

**10. If the ultrasound did not change your management, what is the main reason?**

*Complete only when no management change is considered necessary.*

| ☐ Management already clinically optimised | ☐ Findings insufficiently actionable |
| --- | --- |
| ☐ Ultrasound quality/interpretability insufficient | ☐ Discordant or complex clinical context |

**11. Based on the ultrasound, how would you classify the predominant haemodynamic profile?**

*Complete when the ultrasound supported or would have changed management.*

| ☐ Predominant congestion | ☐ Predominant poor tolerance to fluid removal/underfilling |
| --- | --- |
| ☐ Cardiac dysfunction limiting tolerance | ☐ Mixed profile: congestion plus poor tolerance |
| ☐ Unclear/non-contributory |  |

**12. Which ultrasound domain most influenced your judgement?**

| ☐ Cardiac ultrasound | ☐ Lung ultrasound |
| --- | --- |
| ☐ Venous congestion/VEXUS | ☐ Combination of several domains |

**13. Did the ultrasound results increase your confidence in your therapeutic decision?**

| ☐ No | ☐ Yes, slightly |
| --- | --- |
| ☐ Yes, moderately | ☐ Yes, strongly |

**14. Which aspect(s) of management would you modify on the basis of the ultrasound findings?**

*Select all that apply when a change would have been considered.*

| ☐ Ultrafiltration target | ☐ Dry-weight strategy |
| --- | --- |
| ☐ Session duration | ☐ Dialysate temperature |
| ☐ Sodium prescription/profile | ☐ Antihypertensive adjustment |
| ☐ Diuretic adjustment | ☐ Additional/extra dialysis session |
| ☐ Additional investigations | ☐ Other: __________________________ |

**15. If applicable, what revised ultrafiltration target would you set?**

________________ mL

**16. If applicable, what revised estimated dry weight would you set?**

________________ kg

*This publication version reproduces the questionnaire content described in the manuscript. Nominative physician-identification fields are omitted in accordance with the final data-collection plan.*
